# Supplementary material for: Structures of pseudorabies virus capsids
Source: Nat Commun. 2022 Mar 22;13:1533. doi: 10.1038/s41467-022-29250-3 (PMC8940892; doi:10.1038/s41467-022-29250-3)
Supplement: Supplementary file 1 — Supplementary Information [file 41467_2022_29250_MOESM1_ESM.pdf]

Supplementary Information for

Structures of Pseudorabies Virus Capsids

Guosong Wang [1,2,#], Zhenghui Zha [1,2,#], Pengfei Huang [1,2,#], Hui Sun [1,2,#], Yang Huang [1,2], Maozhou He [1,2], Tian Chen [1,2], Lina Lin [1,2], Zhenqin Chen [1,2], Zhibo Kong [1,2], Yuqiong Que [1,2], Tingting Li [1,2], Ying Gu [1,2], Hai Yu [1,2], Jun Zhang [1,2], Qingbing Zheng [1,2,*], Yixin Chen [1,2,*], Shaowei Li [1,2,*], Ningshao Xia [1,2,3,*]

[1] State Key Laboratory of Molecular Vaccinology and Molecular Diagnostics, School of Public Health, School of Life Sciences, Xiamen University, Xiamen 361102, China

[2] National Institute of Diagnostics and Vaccine Development in Infectious Diseases, Xiamen University, Xiamen 361102, China

[3] Research Unit of Frontier Technology of Structural Vaccinology, Chinese Academy of Medical Sciences, Xiamen 361102, China

[*] Corresponding authors:

E-mail address: abing0811@xmu.edu.cn (Q. Zheng), yxchen2008@xmu.edu.cn (Y. Chen), shaowei@xmu.edu.cn (S. Li) and nsxia@xmu.edu.cn (N. Xia).

[#] These authors contributed equally.

This PDF file includes:

Supplementary Figures 1-14

Supplementary Tables 1-2

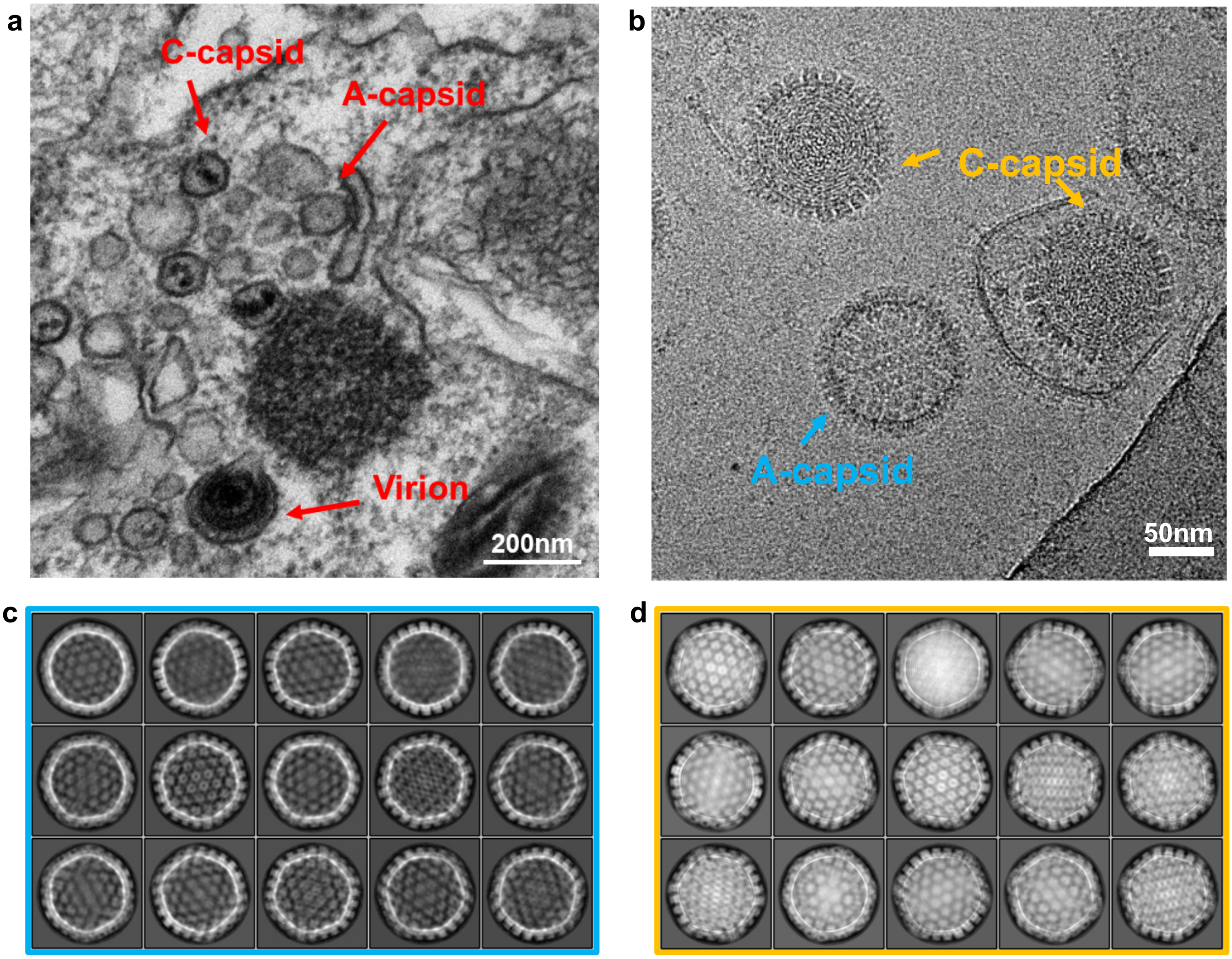

Supplementary Figure 1. Electron microscopy imaging and 2D classification of PRV particles. Representative TEM images of PRV-infected Panc-1 cells **(a)** and cryo-EM micrograph **(b)** and 2D image processing results of PRV A-capsid **(c)** and C-capsid **(d)**. Experiments were performed three times independently with similar results. Scale bars in **a**, **b** denote 200 nm and 50nm respectively.

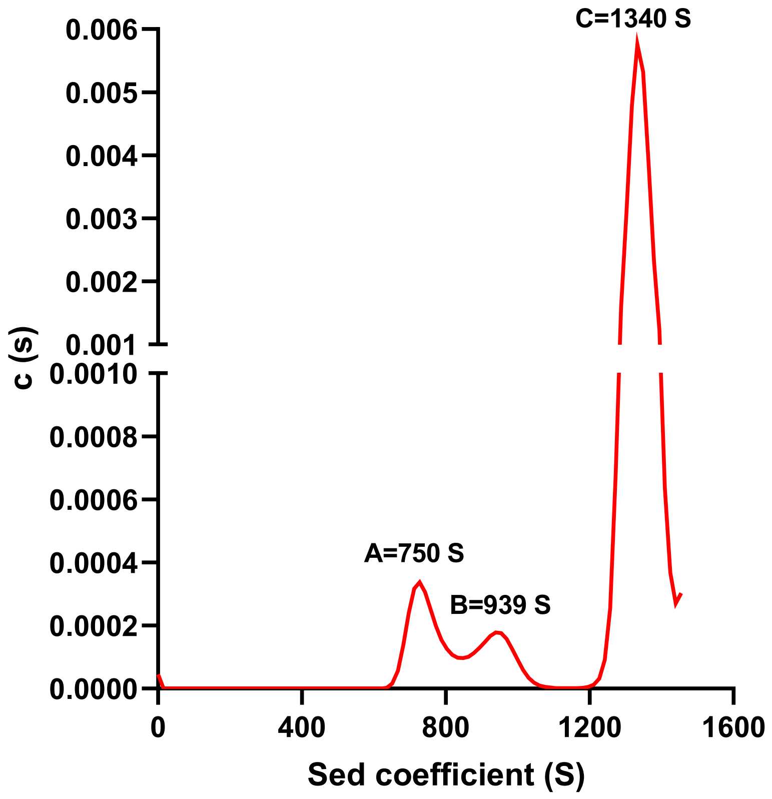

Supplementary Figure 2. Analytical ultracentrifugation analysis of PRV purified particles. The data were analyzed using the program SedFit in c(s) mode to give an apparent distribution of sedimentation coefficients.

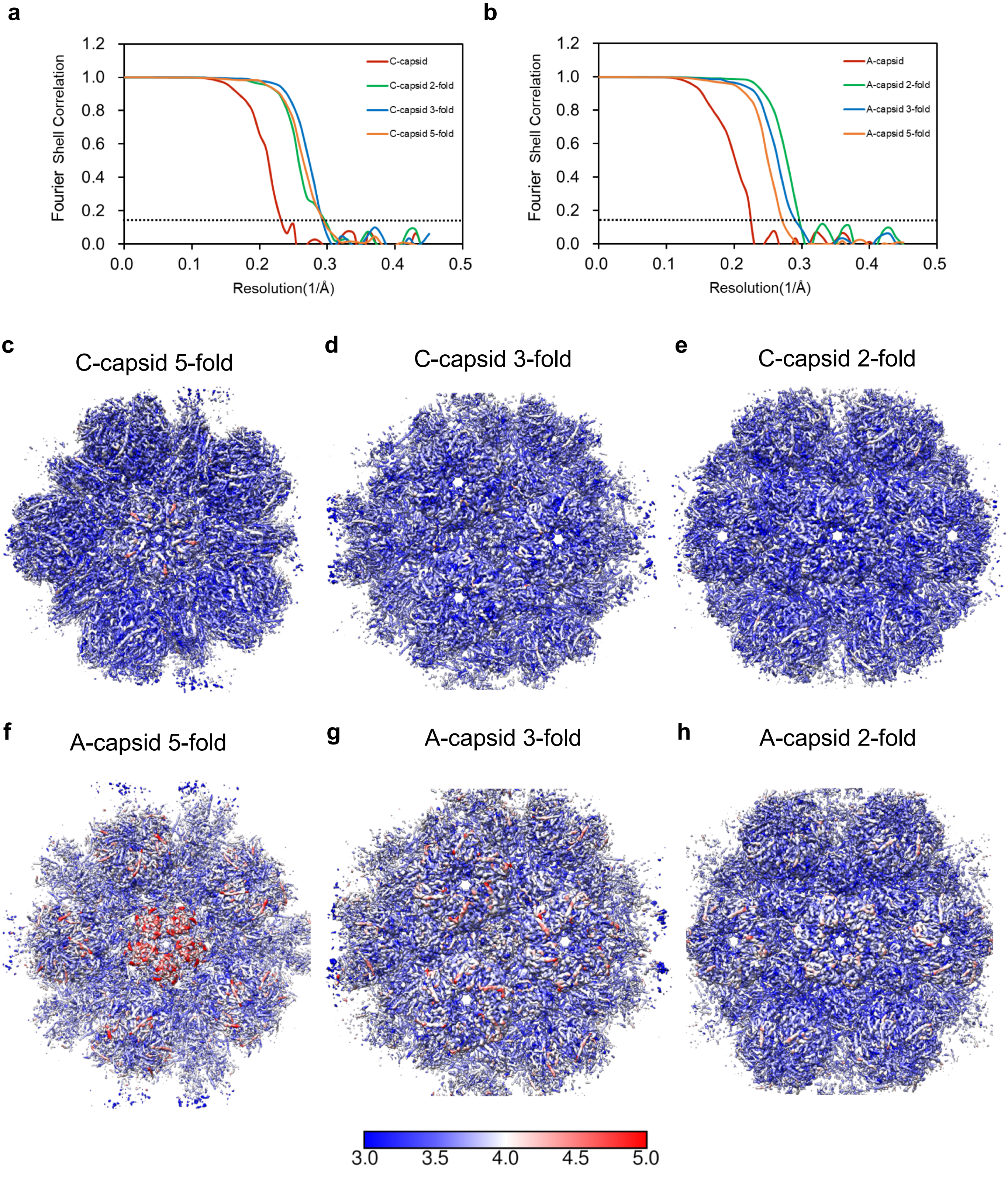

Supplementary Figure 3. Global and local resolution analysis of 3D reconstructions. (a and b) Fourier shell correlation (FSC) of icosahedral and localized 3D reconstructions of C-capsid **(a)** and A-capsid **(b)** were plotted against spatial frequency. **(c-e)** Resmap analysis of the localized reconstructions of the C-capsid at 5- **(c)**, 3- **(d)** and 2-fold **(e)**. **(f-h)** Resmap analysis of the localized reconstructions of the A-capsid at 5- **(f)**, 3- **(g)** and 2-fold **(h)**.

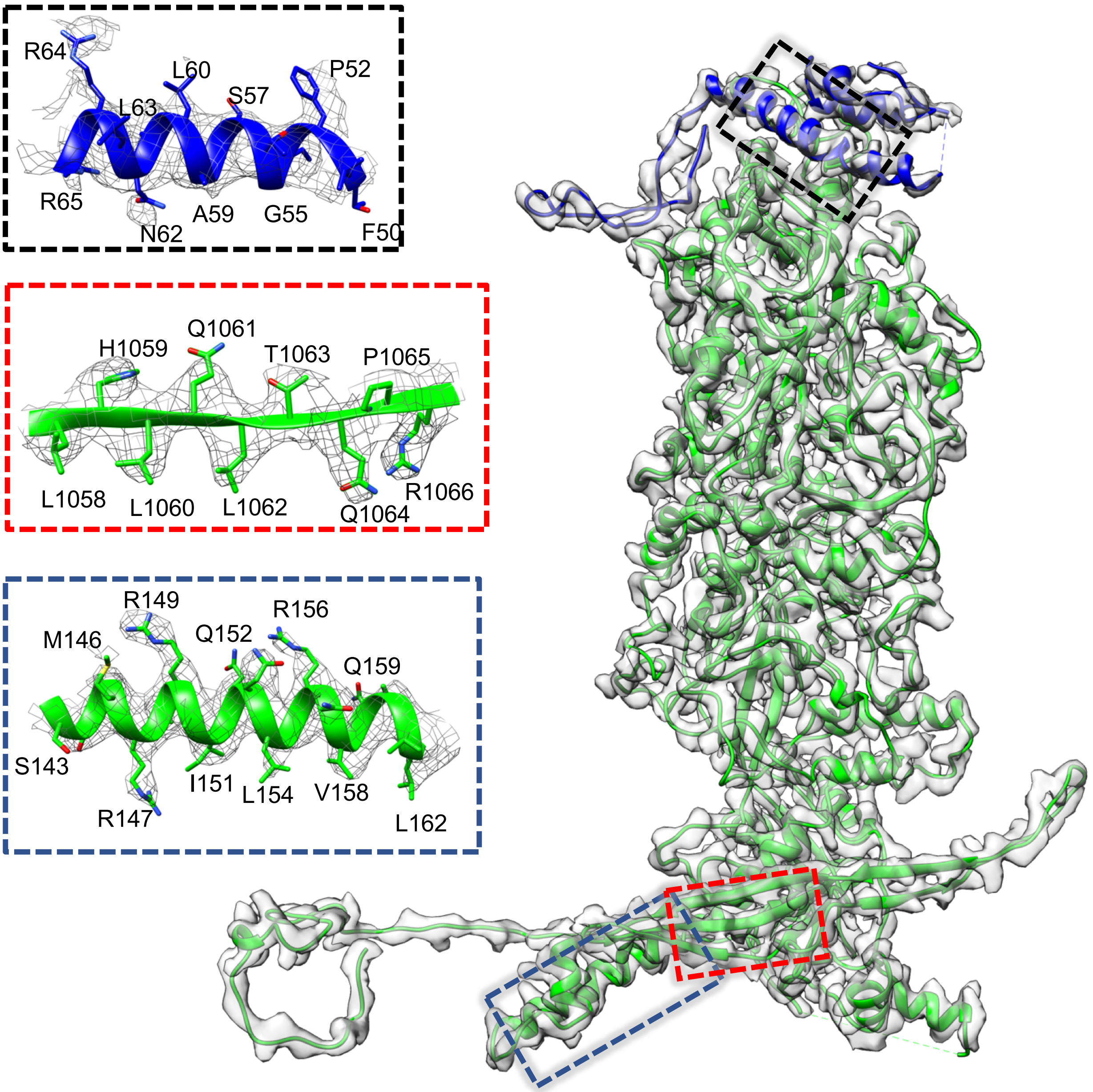

Supplementary Figure 4. Density maps and atomic models of the C5 hexon MCP and C5 SCP. Density map (gray) of C5 hexon MCP and C5 SCP from the 2-fold sub-particle reconstruction of the C-capsid. Boxed regions are enlarged in the colored-edged boxes, with densities shown as a gray mesh and the atomic models as ribbons/sticks.

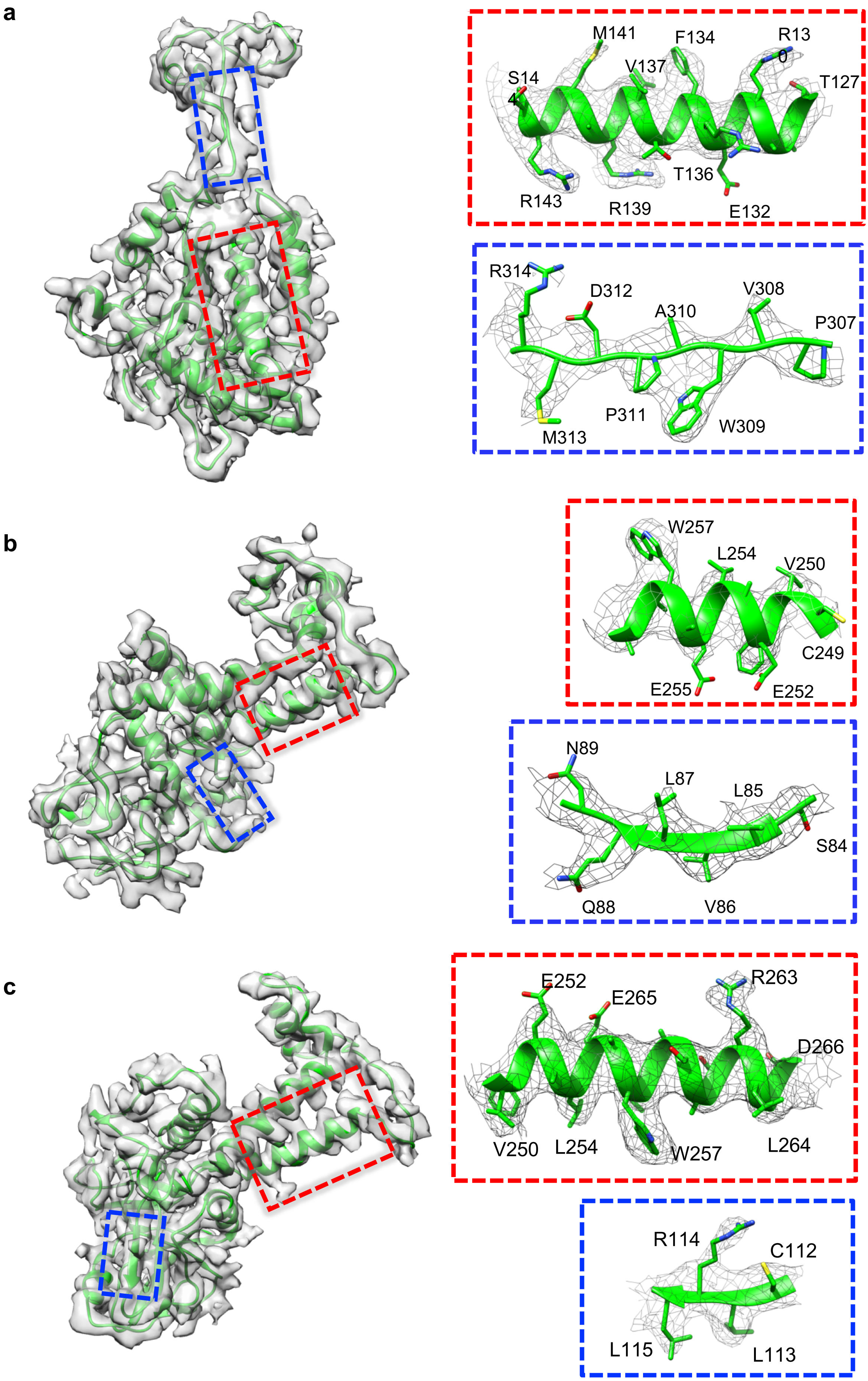

a
M141
F134
R13
0
S14
4
V137
T127
T136
R143
R139
E132
R314
D312
A310
V308
P307
P311
W309
M313
b
W257
L254
V250
C249
E255
E252
N89
L87
L85
S84
Q88
V86
c
E252
E265
R263
D266
V250
L254
W257
L264
R114
C112
L115
L113

Supplementary Figure 5. Density maps and atomic models of Tri1 (a), Tri2a (b) and Tri2b (c) consisting Tb. Density maps (gray) of Tri1 **(a)**, Tri2a **(b)** and Tri2b **(c)** from the 2-fold sub-particle reconstruction of the C-capsid. Boxed regions are enlarged in the colored-edges boxes, with densities shown as a gray mesh and the atomic models as ribbons/sticks.

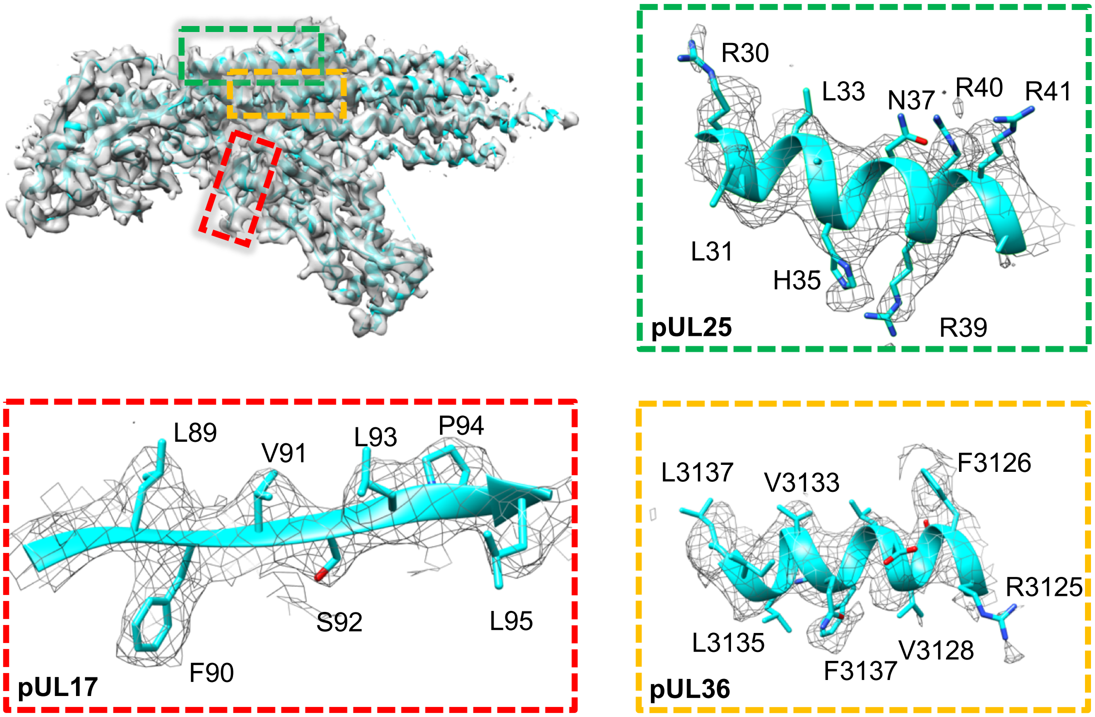

Supplementary Figure 6. Density map and atomic model of CATC. Density map (gray) of CATC from the 5-fold sub-particle reconstruction of C-capsid. Boxed regions are enlarged in the colored-edged boxes, with densities shown as a gray mesh and the atomic models as ribbons/sticks.

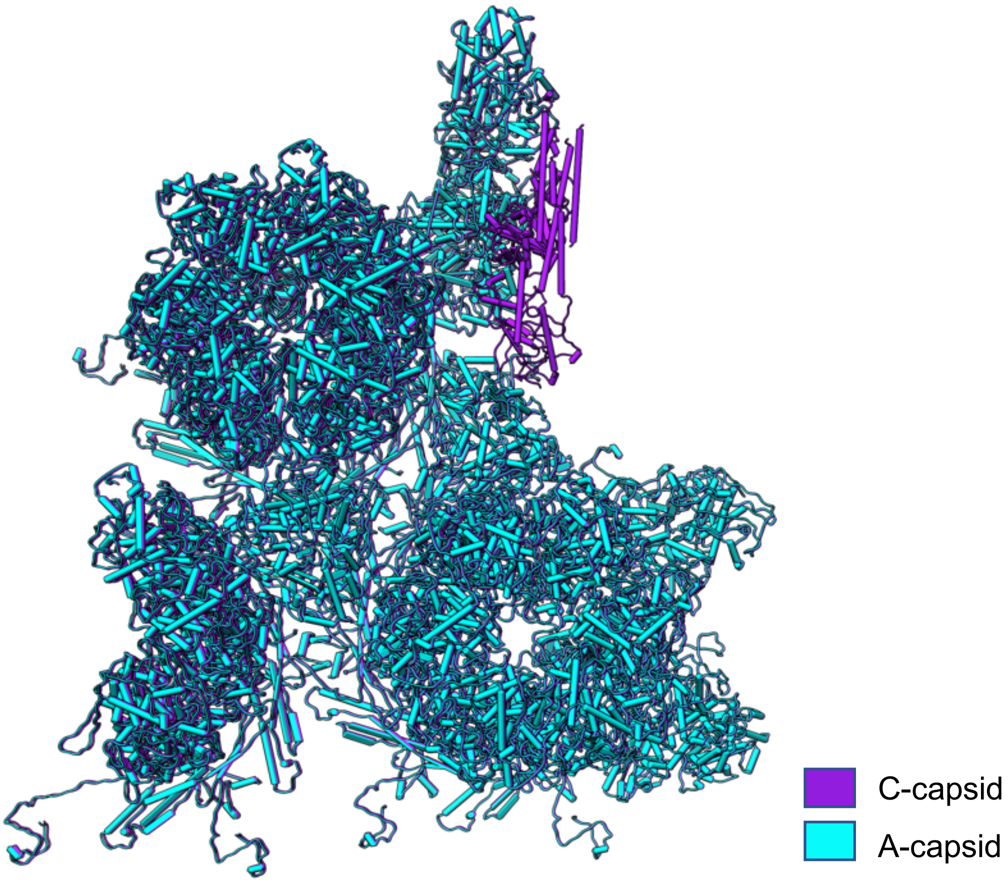

Supplementary Figure 7. Structural comparison of PRV C- and A-capsid. Superposition of the asymmetric units of C-capsid (purple) and A-capsid (cyan) shows highly structural similarity.

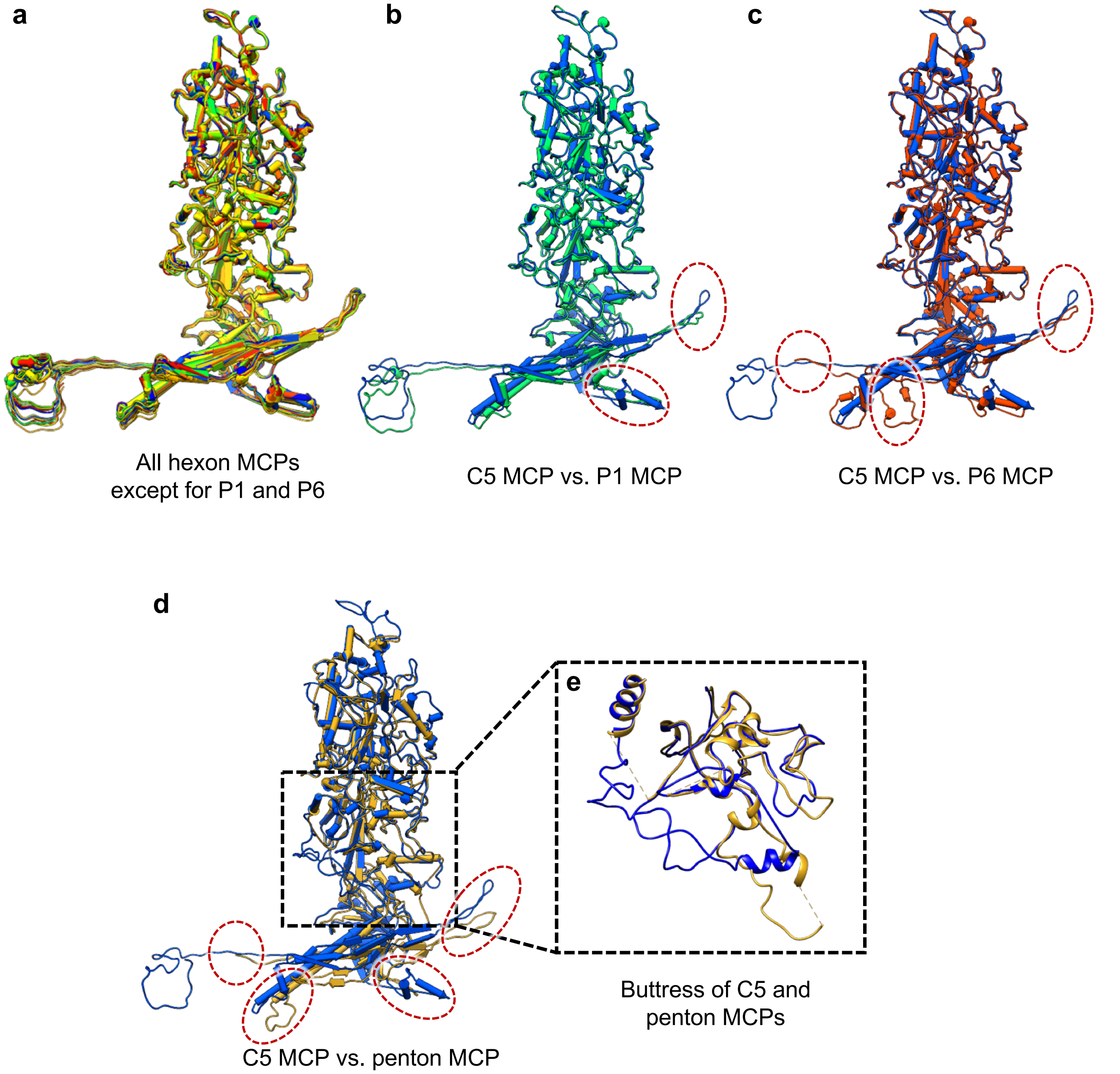

Supplementary Figure 8. Structural comparison of MCPs. (a) Superimposition of all hexon MCPs except for P1 and P6. **(b)** Superimposition of C5 MCP and P1 MCP. **(c)** Superimposition of C5 MCP and P6 MCP. **(d, e)** Superimposition of C5 MCP and penton MCP **(d)** and a close up view of the buttress regions of C5 MCP and penton MCP **(e)**. Red dashed boxes highlight the structural difference between MCPs.

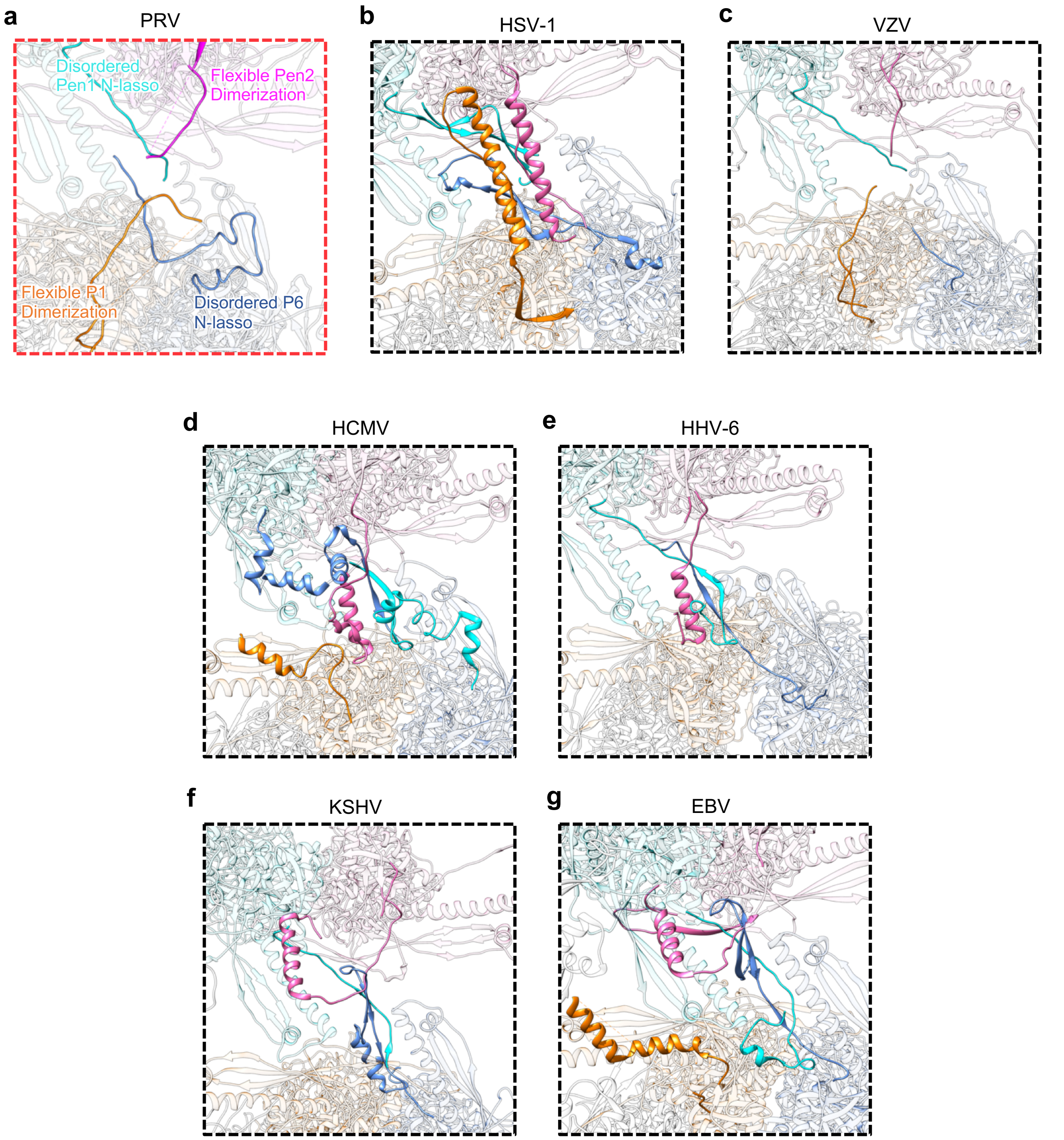

Supplementary Figure 9. Comparisons of the interactions between hexon and penton MCPs in PRV (a), HSV-1 (b), VZV (c), HCMV (d), HHV-6 (e), KSHV (f) and EBV (g). The close-up views of interactions between hexon and penton of each virus are shown as cartoon with color scheme according to Figure 3.

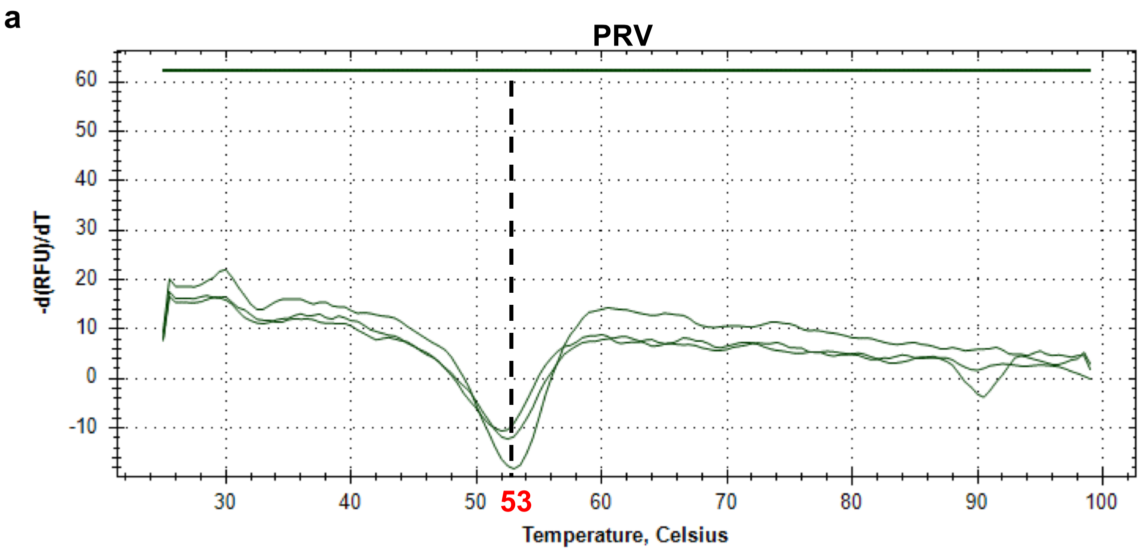

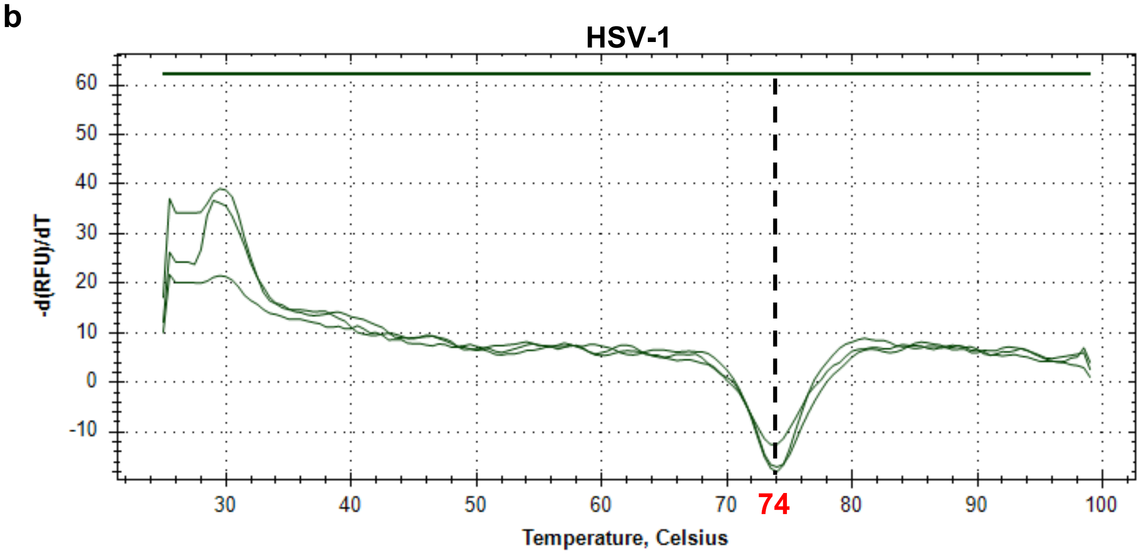

Supplementary Figure 10. Particle stability thermal release assay of purified PRV and HSV-1 capsids. Purified capsids (a mixture of A-, B- and C-capsids) of PRV **(a)** and HSV-1 **(b)** were subjected to the thermofluor stability assay using the dyes SYTO9 to detect DNA exposures, respectively. The average negative first derivative of the fluorescence (measured in triplicate) with respect to temperature (−d(RFU)/dT) is shown.

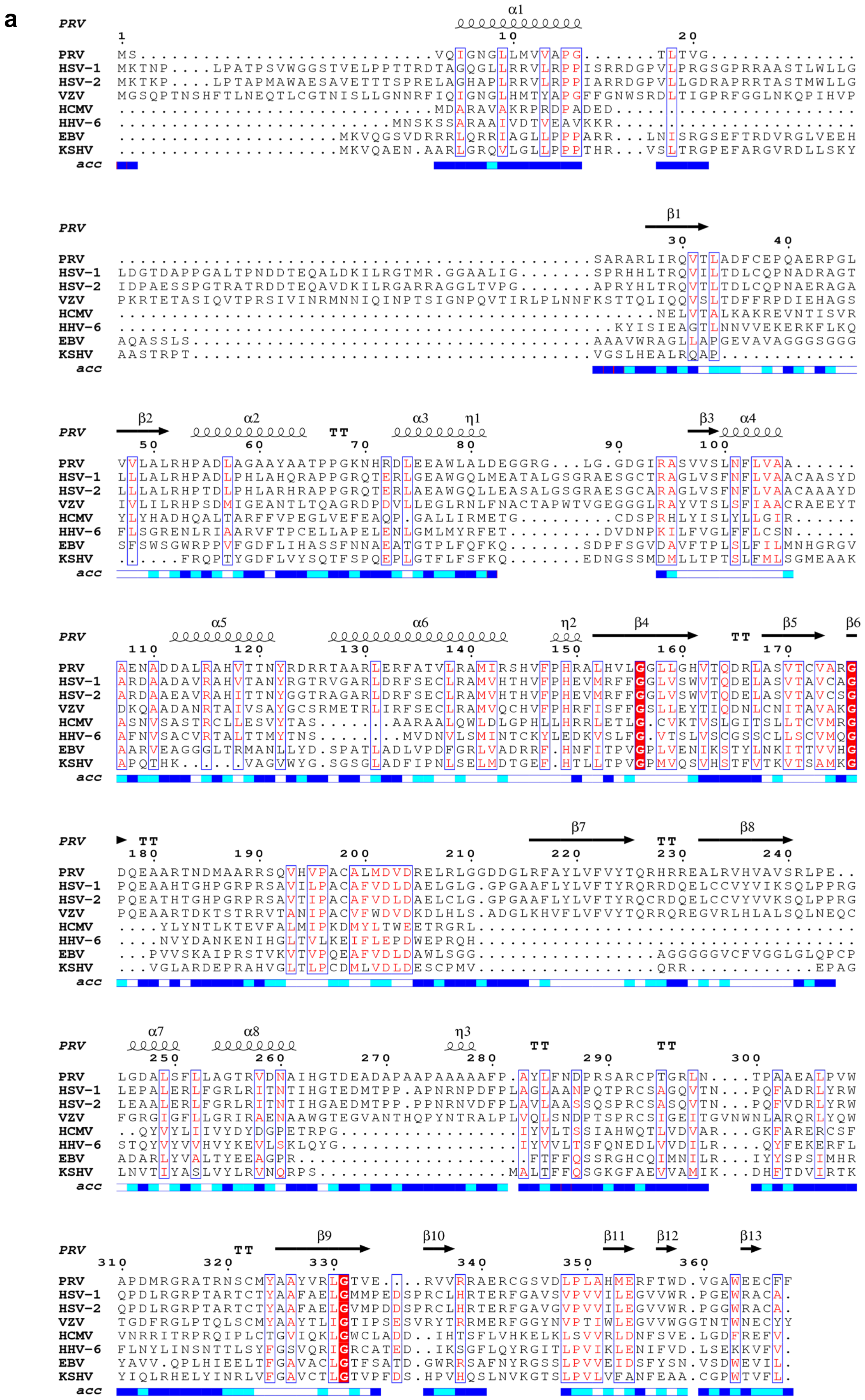

(Continued on next page)

b

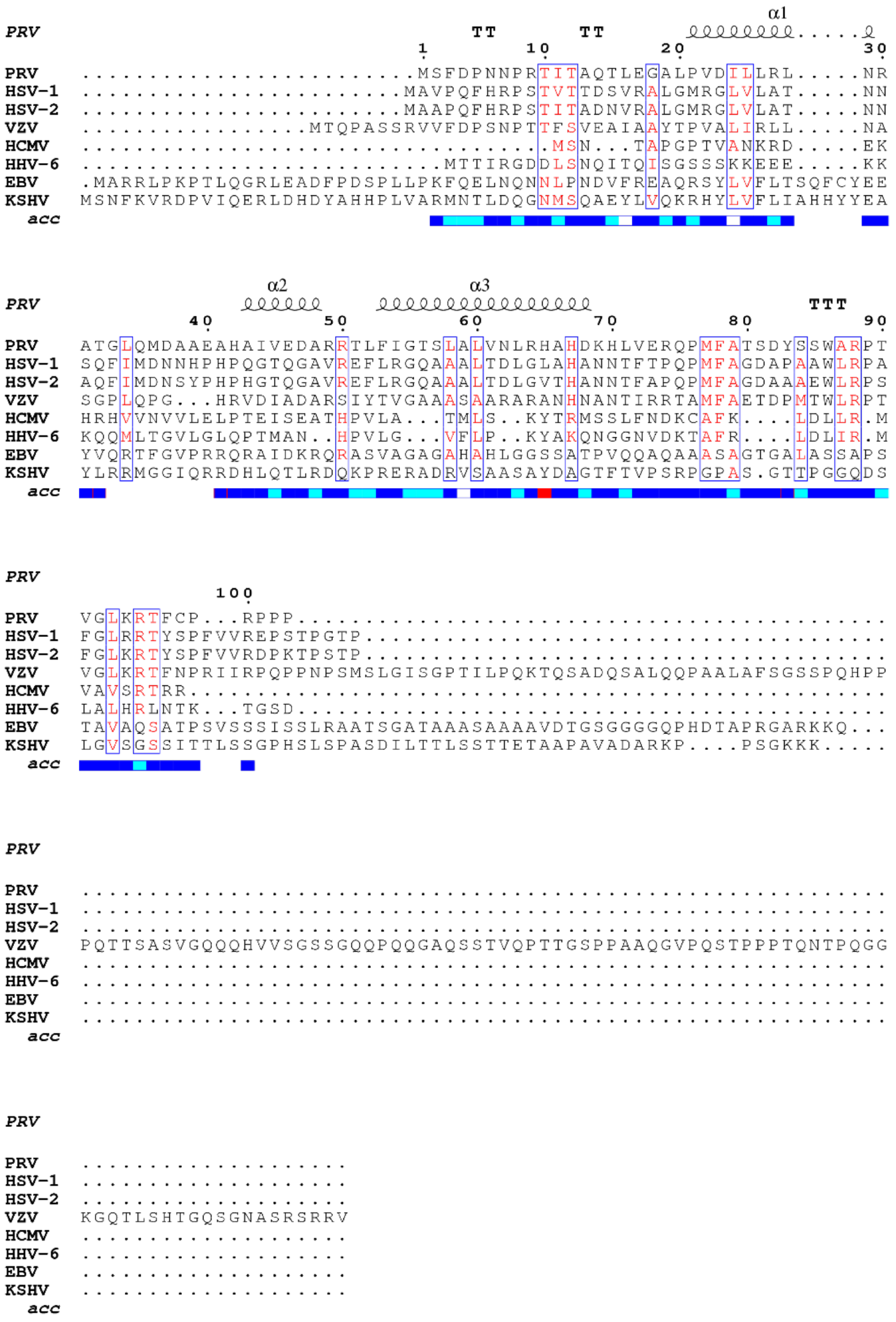

Supplementary Figure 11. Structure-based sequence alignments of representative herpesvirus Tri1s (a) and SCPs (b). Espript representation of a structure-based sequence alignment of SCP and Tri1 sequences of RPV, HSV-1, HSV-2, VZV, HCMV, HHV-6, EBV and KSHV (GenBank accession no. JF797217.1, JQ780693.1, JN561323.2, AB097933.1, GU305914.1, AB021506.1, V01555.2 and GQ994935.1, respectively). Secondary structure elements and relative accessibility are indicated above and below the sequences, respectively.

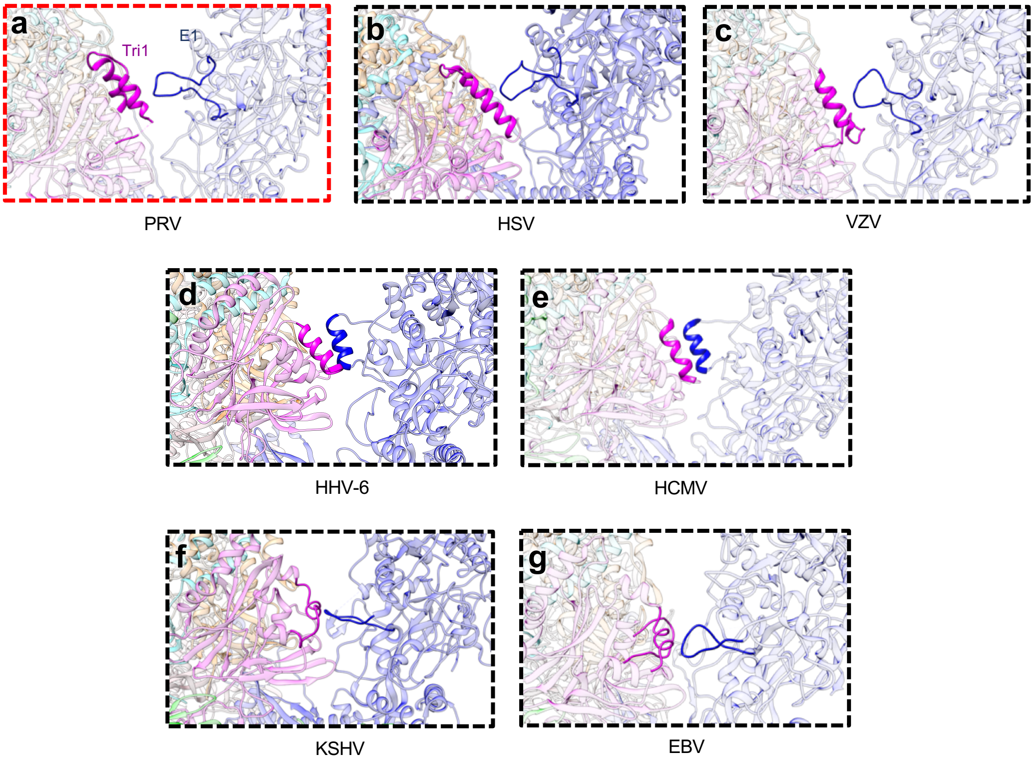

Supplementary Figure 12. Structural comparisons of the interactions of the triplex with surrounding MCPs in PRV (a), HSV (b), VZV (c), HHV-6 (d), HCMV (e), KSHV (f) and EBV (g). The interacting regions between the triplex (Tri1) and MCP (E1 hexon) are highlighted in color of magenta and blue, respectively. Interactions are mediated by the buttress arms of the MCPs that inserting into and interaction with the triplexes.

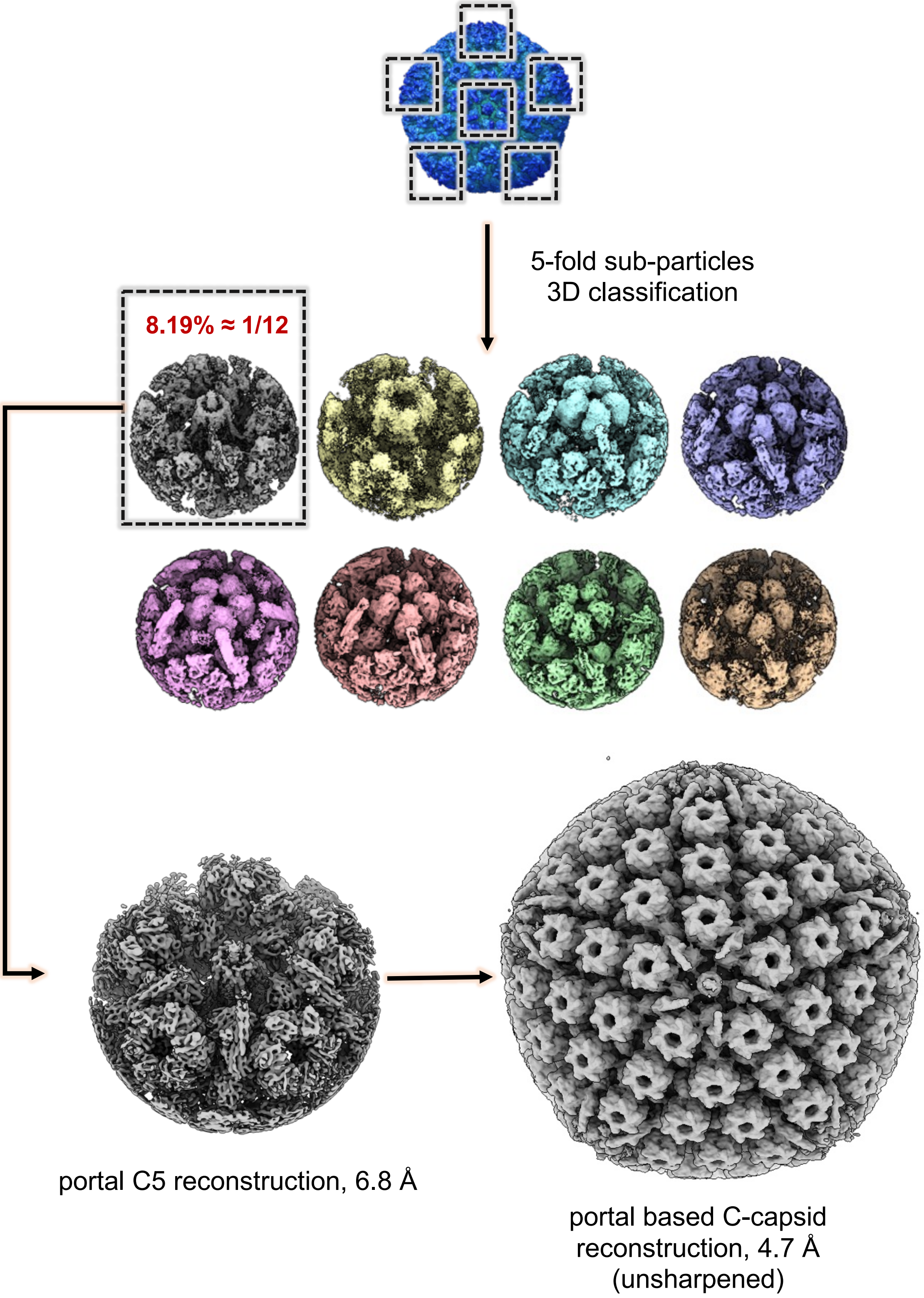

Supplementary Figure 13. Data processing workflow for portal-based sub-particle reconstructions. The 5-fold sub-particles of C-capsids were extracted for 3D classification and the portal vertexes (8.19% of total sub-particles) were classified out for further reconstruction with C5 symmetry (6.8 Å). The portal based whole capsid reconstruction were then performed with C5 symmetry (4.7 Å).

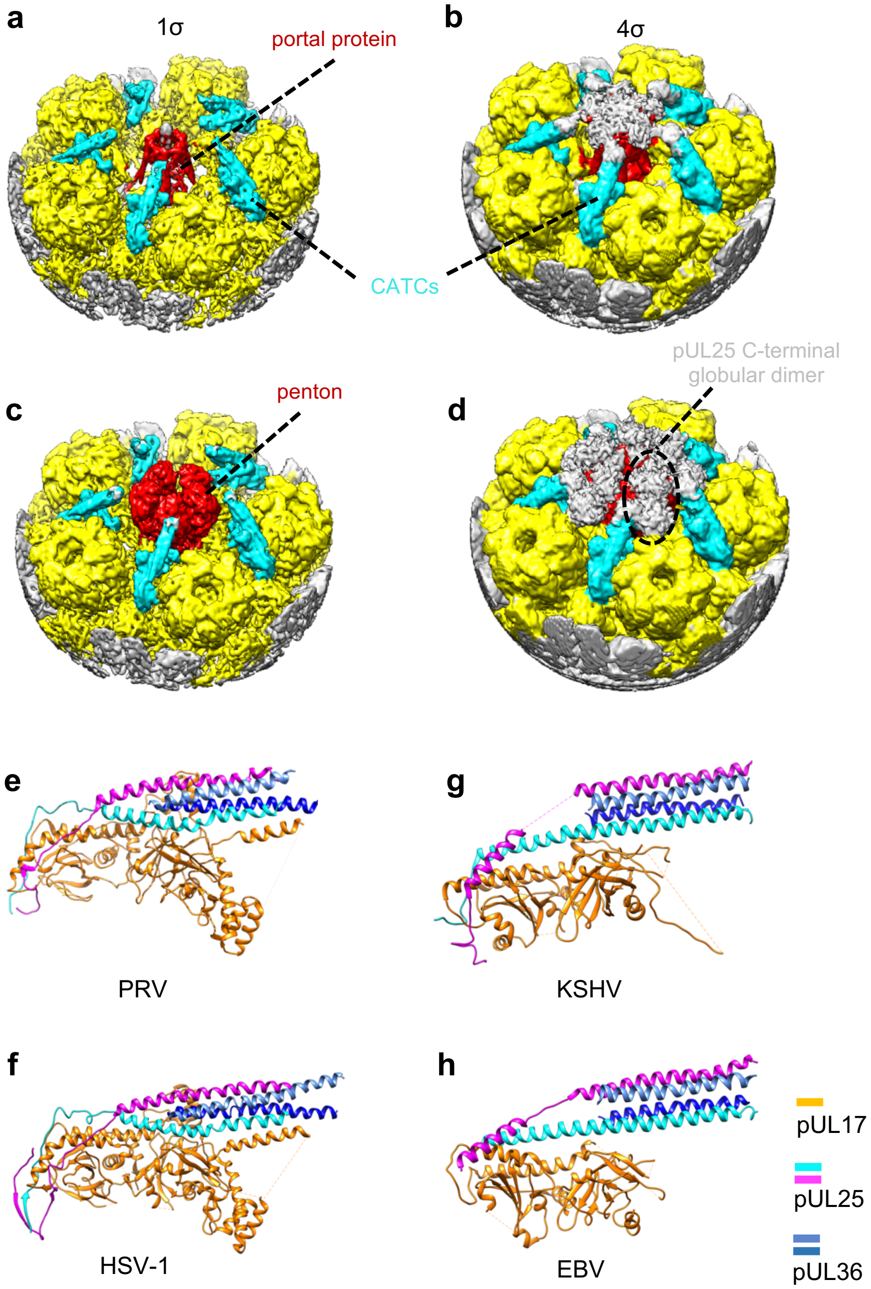

Supplementary Figure 14. Structural comparisons of PRV 5-fold vertexes and the CATCs from different herpesviruses. (a and **b)** PRV portal vertex structures at different thresholds. **(c** and **d)** PRV penton vertex structures at different thresholds. The lower threshold display of the maps show extra density blob of pUL25 C-terminal globular dimer in both portal vertex **(b)** and penton vertex **(d)**. **(e-h)** CARTs structures of α-herpesviruses PRV **(e),** HSV-1 (**f**) and γ-herpesviruses KSHV (**g**) and EBV (**h**).

Supplementary Table 1. Statistics of cryo-EM data collection, 3D reconstructions, model refinement and model validation.

	C-capsid	A-capsid	C-capsid sub 2-fold	C-capsid sub 3-fold	C-capsid sub 5-fold	A-capsid sub 2-fold	A-capsid sub 3-fold	A-capsid sub 5-fold	C-capsid portal	C-capsid with portal
**Data collection and processing**										
Magnification					×93,000					
Voltage (kV)					300					
Electron exposure (e-/Å^2)					25					
Defocus range (μm)					-1.8 to -2.5					
Pixel size (Å)					1.117					
Micrographs (no.)					14,110					
Selected particles (no.)	14,252	8,899	427,560	285,040	171,024	266,970	177,980	106,788	14,022	14,022
Symmetry imposed	I2	I2	C2	C3	C5	C2	C3	C5	C5	C5
Map resolution (Å)	4.43	4.53	3.43	3.46	3.31	3.41	3.5	3.64	6.79	4.72
Map sharpening B factor (Å2)	-90	-90	-90	-90	-90	-90	-90	-90	-90	-90
**Refinement**										
Model composition										
Non-hydrogen atoms	211,439	205,902	/	/	/	/	/	/	/	/
Protein residues	27,399	26,679	/	/	/	/	/	/	/	/
RMSD										
Bond lengths (Å)	0.0047	0.0061	/	/	/	/	/	/	/	/
Bond angles (°)	1.1	1.09	/	/	/	/	/	/	/	/
Validation										
MolProbity score	2.11	2.13	/	/	/	/	/	/	/	/
Clashscore	11.71	11.53	/	/	/	/	/	/	/	/
Poor rotamers (%)	0.13	0.15	/	/	/	/	/	/	/	/
Ramachandran plot										
Favored (%)	90.62	89.74	/	/	/	/	/	/	/	/
Allowed (%)	9.29	10.17	/	/	/	/	/	/	/	/
Disallowed (%)	0.09	0.1	/	/	/	/	/	/	/	/

Supplementary Table 2. Identity percentage of the amino acid sequence of the PRV capsid protein against the homologs of other herpesviruses.

Gene	Encoded protein	HSV-1	HSV-2	VZV	HCMV	HHV-6	EBV	KSHV
UL19	major capsid protein	57.25	57.54	56.94	22.80	23.27	26.08	26.96
UL15	DNA packaging terminase subunit 1	56.30	56.70	52.03	32.89	31.09	29.60	29.29
UL25	DNA packaging tegument protein	48.36	47.95	42.20	17.08	17.28	20.56	19.71
UL18	capsid triplex subunit 2	45.60	45.28	48.73	19.81	14.85	13.55	17.09
UL6	Portal protein	45.37	45.57	42.69	20.79	18.14	23.01	23.58
UL38	capsid triplex subunit 1	35.97	35.04	32.02	14.13	11.88	15.12	15.00
UL17	DNA packaging tegument protein	34.71	35.19	31.91	12.23	8.70	15.75	18.90
UL35	small capsid protein	29.20	27.68	15.97	11.54	4.85	10.80	10.40
UL36	large tegument protein	27.65	28.24	24.43	10.49	8.34	13.64	13.55
