# Supplementary material for: Structures of pseudorabies virus capsids
Source: Nat Commun. 2022 Mar 22;13:1533. doi: 10.1038/s41467-022-29250-3 (PMC8940892; doi:10.1038/s41467-022-29250-3)
Supplement: Supplementary file 3 — Description of Additional Supplementary Files [file 41467_2022_29250_MOESM3_ESM.pdf]

Description of Additional Supplementary Files

File name: Supplementary Movie 1

Description: Cryo-EM reconstruction of PRV C-capsid
