# Supplementary material for: Structures of pseudorabies virus capsids
Source: Nat Commun. 2022 Mar 22;13:1533. doi: 10.1038/s41467-022-29250-3 (PMC8940892; doi:10.1038/s41467-022-29250-3)
Supplement: Supplementary file 5 — Reporting Summary [file 41467_2022_29250_MOESM5_ESM.pdf]

nature portfolio

Corresponding author(s): Shaowei Li

Last updated by author(s): Dec 17, 2021

Reporting Summary

Nature Portfolio wishes to improve the reproducibility of the work that we publish. This form provides structure for consistency and transparency in reporting. For further information on Nature Portfolio policies, see our Editorial Policies and the Editorial Policy Checklist.

Statistics

For all statistical analyses, confirm that the following items are present in the figure legend, table legend, main text, or Methods section.

n/a	Confirmed	
☐	☒	The exact sample size (*n*) for each experimental group/condition, given as a discrete number and unit of measurement
☐	☒	A statement on whether measurements were taken from distinct samples or whether the same sample was measured repeatedly
☒	☐	The statistical test(s) used AND whether they are one- or two-sided<br>*Only common tests should be described solely by name; describe more complex techniques in the Methods section.*
☒	☐	A description of all covariates tested
☒	☐	A description of any assumptions or corrections, such as tests of normality and adjustment for multiple comparisons
☐	☒	A full description of the statistical parameters including central tendency (e.g. means) or other basic estimates (e.g. regression coefficient) AND variation (e.g. standard deviation) or associated estimates of uncertainty (e.g. confidence intervals)
☒	☐	For null hypothesis testing, the test statistic (e.g. *F*, *t*, *r*) with confidence intervals, effect sizes, degrees of freedom and *P* value noted<br>*Give P values as exact values whenever suitable.*
☒	☐	For Bayesian analysis, information on the choice of priors and Markov chain Monte Carlo settings
☒	☐	For hierarchical and complex designs, identification of the appropriate level for tests and full reporting of outcomes
☒	☐	Estimates of effect sizes (e.g. Cohen's *d*, Pearson's *r*), indicating how they were calculated

Our web collection on statistics for biologists contains articles on many of the points above.

Software and code

Policy information about availability of computer code

Data collection	The commercial software EPU (Version 1.11, Themor Fisher, https://www.fei.com/software/epu/) was used for cryoEM data collection on FEI Tecnai F30 ((Thermo Fisher Scientific).
Data analysis	All software used for data analysis in this study were available online:<br>1. MotionCor2 (http://msg.ucsf.edu/em/software/motioncor2.html): micrography collection;<br>2. Gctf (https://en.wikibooks.org/w/index.php?title=Software_Tools_For_Molecular_Microscopy&stable=0#Gctf): ctf estimation;<br>3. CisTEM1.0.1 (https://cistem.org/): particle picking and 3D reconstruction;<br>4. Scipion2 (http://scipion.i2pc.es/): subparticle generation;<br>5. Relion3.0 (http://www2.mrc-lmb.cam.ac.uk/relion): CryoEM map reconstruction;<br>6. ResMap (http://resmap.sourceforge.net): CryoEM map resolution estimation;<br>7. Chimera (http://www.cgl.ucsf.edu/chimera): Density maps or structural models based visualization, segmentation and movies generation;<br>8. Accelrys Discovery Studio 4.5 (https://www.3dsbiovia.com): homology modeling;<br>9. Coot0.8.9.1 (http://www2.mrc-lmb.cam.ac.uk/personal/ pemsley/coot): model building;<br>10. Phenix1.14 (http://phenix-online.org): model refinement.<br>11. Molprobity (http://molprobity.biochem.duke.edu): model validation and statistics;<br>12. Clustal Omega (https://www.ebi.ac.uk/Tools/msa/clustalo/): structural-based sequence alignment;<br>13. Pymol2.3 (http://www.pymol.org): Figure generation;<br>14. SEDFIT (http://www.analyticalultracentrifugation.com): Interference sedimentation coefficient distributions calculation.

For manuscripts utilizing custom algorithms or software that are central to the research but not yet described in published literature, software must be made available to editors and reviewers. We strongly encourage code deposition in a community repository (e.g. GitHub). See the Nature Portfolio guidelines for submitting code & software for further information.

Data

Policy information about availability of data

All manuscripts must include a data availability statement. This statement should provide the following information, where applicable:

- Accession codes, unique identifiers, or web links for publicly available datasets
- A description of any restrictions on data availability
- For clinical datasets or third party data, please ensure that the statement adheres to our policy

Structure coordinates are deposited in the Protein Data Bank under accession codes 7FJ3 (A-capsid) and 7FJ1 (C-capsid). The corresponding EM density maps have been deposited in the Protein Data Bank under accession numbers EMD-31612 (icosahedral reconstruction of the A-capsid), EMD-31611 (icosahedral reconstruction of the C-capsid), EMD-31610 (portal vertex), EMD-31616 (portal based reconstruction of the C-capsid), EMD-31593 (2-fold sub-particle reconstruction of A-capsid), EMD-31592 (3-fold sub-particle reconstruction of A-capsid), EMD-31591 (5-fold sub-particle reconstruction of A-capsid), EMD-31609 (2-fold sub-particle reconstruction of C-capsid), EMD-31608 (3-fold sub-particle reconstruction of C-capsid) and EMD-31594 (5-fold sub-particle reconstruction of C-capsid).

Field-specific reporting

Please select the one below that is the best fit for your research. If you are not sure, read the appropriate sections before making your selection.

☒ Life sciences ☐ Behavioural & social sciences ☐ Ecological, evolutionary & environmental sciences

For a reference copy of the document with all sections, see nature.com/documents/nr-reporting-summary-flat.pdf

Life sciences study design

All studies must disclose on these points even when the disclosure is negative.

Sample size	Sample sizes were estimated on the basis of previous study (DOI: 10.1038/s41564-020-0785-y).
Data exclusions	No data were excluded from the analyses.
Replication	Reconstructions are the result of calculations performed on the collected data set. Therefore, all reconstructions are reproducible if the workflow is maintained.
Randomization	During 3D refinements, particles were randomly split into two halves then reconstructed two half maps in order to calculate FSC curves in Fourier space.
Blinding	Blinding is not applied in this study as this study is mainly focusing on structural analyses to multiple obtained cryo-EM density maps.

Reporting for specific materials, systems and methods

We require information from authors about some types of materials, experimental systems and methods used in many studies. Here, indicate whether each material, system or method listed is relevant to your study. If you are not sure if a list item applies to your research, read the appropriate section before selecting a response.

Materials & experimental systems

n/a	Involved in the study
☒	☐ Antibodies
☐	☒ Eukaryotic cell lines
☒	☐ Palaeontology and archaeology
☒	☐ Animals and other organisms
☒	☐ Human research participants
☒	☐ Clinical data
☒	☐ Dual use research of concern

Methods

n/a	Involved in the study
☒	☐ ChIP-seq
☒	☐ Flow cytometry
☒	☐ MRI-based neuroimaging

Eukaryotic cell lines

Policy information about cell lines

Cell line source(s)	PK-15 and Panc-1 cell lines were obtained from the American Type Culture Collection.
Authentication	The cell lines were authenticated by the providers through morphology, karyotyping and PCR-based approaches. After

Authentication	receipt of the cell lines, visual inspection of their cellular morphology in culture was routinely performed but no further authentication was conducted in our lab.
Mycoplasma contamination	The cell lines were tested to be negative for mycoplasma contamination prior to experiments.
Commonly misidentified lines (See ICLAC register)	No commonly misidentified cell lines were used in this study.
